# A bias field correction workflow based on generative adversarial network for abdominal cancers treated with 0.35T MR‐LINAC

**DOI:** 10.1002/acm2.70448

**Published:** 2026-01-02

**Authors:** Ching‐Ching Yang, Hung‐Te Yang

**Affiliations:** ^1^ Department of Medical Imaging and Radiological Sciences Kaohsiung Medical University Kaohsiung Taiwan; ^2^ Department of Medical Research Kaohsiung Medical University Hospital Kaohsiung Taiwan; ^3^ Department of Radiation Oncology Kaohsiung Municipal Siaogang Hospital Kaohsiung Taiwan

**Keywords:** bias field artifacts, generative adversarial network, 0.35T MR‐LINAC

## Abstract

**Purpose:**

In this study, a bias field correction workflow was proposed to improve the flexibility and generalizability of the generative adversarial network (GAN) model for abdominal cancer patients treated with a 0.35T magnetic resonance imaging linear accelerator (MR‐LINAC) system.

**Methods:**

Model training was performed using brain MR images acquired on a 3T diagnostic scanner, while model testing was performed using abdominal MR images obtained using a 0.35T MR‐LINAC system. The performance of the proposed workflow was first compared with the GAN model using root‐mean‐square error (RMSE), peak signal‐to‐noise ratio (PSNR), and structural similarity index measure (SSIM). To assess the impact of the workflow on image segmentation, it was also compared with the N4ITK algorithm. Segmentation was performed using the k‐means clustering algorithm with three clusters corresponding to air, fat, and soft tissue. Segmentation accuracy was then evaluated using the Dice similarity coefficient (DSC).

**Results:**

The RMSE values were 30.59, 12.06, 10.37 for the bias field‐corrupted images (I_IN_), GAN‐corrected images (I_GAN_), and images corrected with the proposed workflow (I_OUT_), respectively. Corresponding PSNR values were 42.34, 46.04, 47.04 dB, and SSIM values were 0.84, 0.96, 0.98. For segmentation accuracy, the mean DSC for air masks was 0.95, 0.97, and 0.97; for fat masks, 0.61, 0.71, and 0.74; and for soft tissue masks, 0.60, 0.68, and 0.69, corresponding to I_IN_, N4ITK‐corrected images (I_N4ITK_), and I_OUT_, respectively

**Conclusion:**

By effectively mitigating bias field artifacts, the proposed workflow has the potential to strengthen the clinical utility of MRI‐guided adaptive radiotherapy for abdominal cancers, ensuring safer and more accurate radiation delivery.

## 1 INTRODUCTION

Cone‐beam computed tomography (CBCT) is the most widely utilized onboard imaging modality for target localization in contemporary radiation therapy.^1^, ^2^, ^3^ Its popularity stems from broad availability, relatively low imaging dose, and seamless integration with linear accelerator (LINAC) platforms. However, a fundamental limitation of CBCT is its poor soft‐tissue contrast, which restricts the ability to clearly differentiate tumor boundaries from surrounding normal structures. This limitation is particularly pronounced in anatomical sites where target and organ‐at‐risk (OAR) interfaces are subtle, such as in the abdomen and pelvis. As a result, reliance on CBCT for image guidance can hinder accurate structure delineation, compromise the fidelity of treatment adaptation, and reduce the overall robustness of the adaptive radiotherapy workflow. Magnetic resonance imaging (MRI) offers notably superior soft‐tissue contrast over CBCT, significantly enhancing the precision of target localization and OAR delineation.^4^, ^5^, ^6^ Consequently, MRI‐guided radiotherapy has the potential to improve treatment delivery accuracy, reduce uncertainties, and ultimately optimize the therapeutic ratio in the management of complex and mobile tumors. Despite these advantages, MRI‐based image guidance is not without challenges. One prominent issue is the bias field artifacts, characterized by smooth, low‐frequency variations in signal intensity that result in non‐uniform intensity distributions.^7^, ^8^, ^9^, ^10^ This artifact may obscure structures of interest, reduce image contrast, and compromise quantitative image analysis. Its sources include radiofrequency coil sensitivity variations, magnetic field inhomogeneities, and patient‐related factors. In the context of radiotherapy, bias field artifacts pose a critical problem for automated and semi‐automated segmentation of target and OAR, as errors at this stage can propagate into treatment planning and adaptation, potentially reducing treatment accuracy.^11^, ^12^, ^13^ Deep learning has recently gained significant traction as a powerful tool for compensating image artifacts in medical imaging, owing to its ability to learn complex spatial priors and intensity distributions directly from large‐scale datasets.^14^, ^15^, ^16^ In this study, a bias field correction workflow based on a generative adversarial network (GAN) was proposed for abdominal cancer patients treated with a 0.35T magnetic resonance imaging linear accelerator (MR‐LINAC) system. Generally, larger and more diverse datasets enable a GAN to capture a wider range of patterns and features within the real data distribution. With more examples to learn from, the generator can produce higher‐quality and more diverse outputs, while the discriminator benefits from a richer set of real samples to better distinguish authentic images from generated ones. However, compared with diagnostic MRI scanners operating in the 1.5 to 3T range, which are widely available in clinical practice and serve as the standard for high‐resolution imaging, 0.35T MR‐LINAC systems remain considerably less prevalent due to their more recent introduction, specialized application in image‐guided radiotherapy, and higher cost and infrastructure requirements.^17^, ^18^ Therefore, rather than depending on large‐scale training datasets, a workflow was introduced to enhance the GAN model's flexibility and generalizability. By effectively mitigating bias field artifacts, our proposed method aimed to enhance MR image quality, increase the accuracy and reliability of contour delineation, and ultimately strengthen the clinical utility of MRI‐guided adaptive radiotherapy.

## 2 METHODS

### 2.1 Bias field correction

**FIGURE 1:**
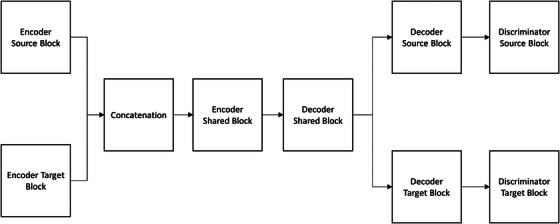
The block diagram of GAN model.

**FIGURE 2:**
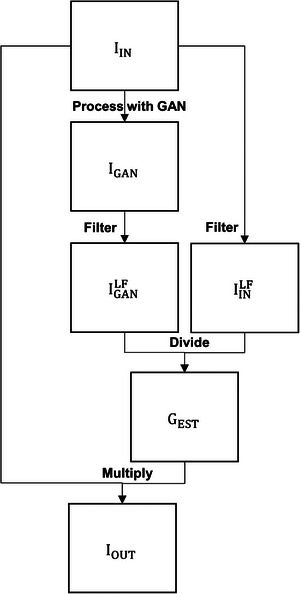
The workflow of the proposed biased field correction method.

Figure 1 depicts the architecture of the GAN model, which comprised two generators and two discriminators.^19^ The generators followed an encoder‐decoder design, with one network dedicated to the bias field‐corrupted domain and the other to the bias field‐compensated domain. Each encoder included two downsampling blocks and five residual blocks, of which two were shared across domains to enforce a common latent representation. The decoders mirrored this structure, and a shared latent encoder block with Kullback‐Leibler (KL) regularization was incorporated to further constrain the latent space. For adversarial training, two PatchGAN discriminators were employed, one for each domain. Each discriminator consisted of four convolutional downsampling blocks with a kernel size of three. Table 1 summarizes the key parameters used across all blocks of the GAN model. The generator objective function combined adversarial, self‐reconstruction, cycle‐consistency, and KL losses in a weighted manner. Model optimization was carried out using the Adam algorithm with a learning rate of 0.0001, batch size of one, weight decay of 0.0001, over 100 epochs. Based on our experience, limited dataset size can hinder a GAN model's ability to accurately synthesize fine image details. To address this limitation, the workflow illustrated in Figure 2 was proposed to improve the model's flexibility and generalizability. First, a bias field‐corrupted image (I_IN_) was processed with the GAN model. Next, both I_IN_ and the GAN‐processed image (I_GAN_) were filtered with a Gaussian lowpass filter to extract the low frequency components in I_IN_ ($I_{\mathrm{IN}}^{\mathrm{LF}}$) and I_GAN_ ($I_{\mathrm{GAN}}^{\mathrm{LF}}$). An estimated gradient map (G_EST_) was then generated by dividing $I_{\mathrm{GAN}}^{\mathrm{LF}}$ with $I_{\mathrm{IN}}^{\mathrm{LF}}$. Finally, I_IN_ was multiplied by G_EST_ to compensate for the bias field, resulting the final output image (I_OUT_).

**TABLE 1:** Key parameters used in the GAN model.

Block	Layer / Component	Key Parameters
Encoder Source	Input	Size: [256 256 1]
	First Convolution	Filters: 64, Filter size: 7×7, Stride: 1, Activation: Leaky ReLU (0.2), No normalization
	Downsampling	Number: 2, Filter size: 3×3, Stride: 2, Normalization: InstanceNorm, Activation: ReLU
	Private Residual	Number: 3, Filter size: 3×3, Stride: 1, Normalization: InstanceNorm, Activation: ReLU, Skip connections
	Shared Residual	Number: 2, Filter size: 3×3, Stride: 1, Normalization: InstanceNorm, Activation: ReLU, Skip connections
	Padding	Symmetric
	Weight Initialization	He initialization
	Output	Feature map to shared encoder
Encoder Target	Input	Size: [256 256 1]
	First Convolution	Filters: 64, Filter size: 7×7, Stride: 1, Activation: Leaky ReLU (0.2), No normalization
	Downsampling	Number: 2, Filter size: 3×3, Stride: 2, Normalization: InstanceNorm, Activation: ReLU
	Private Residual	Number: 3, Filter size: 3×3, Stride: 1, Normalization: InstanceNorm, Activation: ReLU, Skip connections
	Shared Residual	Number: 2, Filter size: 3×3, Stride: 1, Normalization: InstanceNorm, Activation: ReLU, Skip connections
	Padding	Symmetric
	Weight Initialization	He initialization
	Output	Feature map to shared encoder
Encoder Shared	Residual Layers	Number: 2, Filter size: 3×3, Stride: 1, Normalization: InstanceNorm, Activation: ReLU, Skip connections
	Padding	Symmetric
	Weight	He initialization
	Output	Shared latent feature map
Decoder Shared	Residual Layers	Number: 2, Filter size: 3×3, Stride: 1, Normalization: InstanceNorm, Activation: ReLU, Skip connections
	Padding	Symmetric
	Weight Initialization	He initialization
	Output	Feature map to private decoders
Decoder Source	Upsampling	Number: 2, Filter size: 3×3, Stride: 2, Method: TransposedConv or Bilinear, Normalization: InstanceNorm, Activation: ReLU
	Final Convolution	Filters: Output channels (= 1), Filter size: 7×7, Stride: 1, Activation: Tanh
	Padding	Symmetric
	Weight Initialization	He initialization
	Output	Reconstructed source‐domain image
Decoder Target	Upsampling	Number: 2, Filter size: 3×3, Stride: 2, Method: TransposedConv or Bilinear, Normalization: InstanceNorm, Activation: ReLU
	Final Convolution	Filters: Output channels (= 1), Filter size: 7×7, Stride: 1, Activation: Tanh
	Padding	Symmetric
	Weight Initialization	He initialization
	Output	Reconstructed target‐domain image
Discriminator Source	Input	Size: [256 256 1]
	Downsampling Blocks	Number: 4, Filter size: 3×3, Stride: 2, Activation: Leaky ReLU, No normalization
	Weight Initialization	Narrow‐normal
	Padding	Symmetric
	Output	PatchGAN feature map for source‐domain discrimination
Discriminator Target	Input	Size: [256 256 1]
	Downsampling Blocks	Number: 4, Filter size: 3×3, Stride: 2, Activation: Leaky ReLU, No normalization
	Weight Initialization	Narrow‐normal
	Padding	Symmetric
	Output	PatchGAN feature map for target‐domain discrimination

### 2.2 Training datasets

Model training was performed with T1‐weighted magnetization‐prepared rapid gradient‐echo (MPRAGE) MR images acquired on a 3T Siemens MAGNETOM Prisma scanner (Siemens Healthcare GmbH, Erlangen, Germany). The dataset, initially used by Yaakub et al. for evaluating their MR‐to‐CT image translation model, was accompanied by pseudo CT images synthesized by their network.^20^ The corresponding dataset, sub‐test01_t1w.nii and sub‐test01_pct.nii, were available at https://github.com/sitiny/mr‐to‐pct. The images were acquired in the sagittal plane using the following parameters: repetition time (TR) of 2100 ms, echo time (TE) of 2.26 ms, inversion time (TI) of 900 ms, flip angle (FA) of 8°, GRAPPA (GeneRalized Autocalibrating Partial Parallel Acquisition) acceleration factor of 2, and a voxel size of 1 mm^3^. The MR images were preprocessed according to the following steps: (1) bias field correction of the T1‐weighted MR images using the N4ITK algorithm; (2) application of binary head mask to the MR images to suppress noise outside the head region; and (3) resampling of the MR images to 1 mm^3^ isotropic resolution, resulting in a final matrix size of 208 × 288 × 288. To simulate bias field artifacts, 19 inhomogeneity patterns were generated and applied to the MR data, producing a total of 5,472 input‐label image pairs for model training.

### 2.3 Testing datasets

Model testing was performed using abdominal scans from the SynthRAD2025 Grand Challenge acquired on a 0.35T ViewRay MRIdian MR‐LINAC system (ViewRay, Mountain View, California, USA) with a balanced steady‐state free‐precession (bSSFP) sequence.^21^ The acquisition parameters included a TE of approximately 1.27–1.62 ms, TR of 3.0–3.8 ms, a flip angle of 60°, in‐plane voxel spacing of roughly 1.5–1.6 mm, and a slice thickness of 3 mm. The on‐board MR images acquired for daily treatment were paired with corresponding planning CT images obtained on an Aquilion LB CT scanner (Canon Medical Systems, Otawara, Japan). The CT acquisition parameters included a tube voltage of 120 kVp, tube current of approximately 40–420 mA, a slice thickness of 3 mm, and an in‐plane pixel spacing ranging from about 0.6–1.4 mm. Twelve cases with pronounced bias field artifacts were analyzed, corresponding to patient identification numbers (ID#): 1ABB002, 1ABB024, 1ABB036, 1ABB066, 1ABB073, 1ABB077, 1ABB084, 1ABB086, 1ABB098, 1ABB117, 1ABB150, and 1ABB151. The datasets are publicly available at http://zenodo.org/record/14918089. The image pairs were preprocessed as follows: (1) MR images were rigidly registered to their corresponding planning CT images using the Elastic registration framework; (2) all images were resampled to a uniform voxel spacing of 1 × 1 × 3 mm^3^; (3) and an outline mask was generated for each patient case.

### 2.4 Model evaluation

First, the performance of the proposed workflow was evaluated against the GAN model using root‐mean‐square error (RMSE), peak signal‐to‐noise ratio (PSNR), and structural similarity index measure (SSIM). RMSE is mathematically expressed as follows:

(1) $$\mathrm{RMSE}\;=\sqrt{\frac{\sum_{i=1}^{V}{\left(I_{\mathrm{TEST}}-I_{\mathrm{LABEL}}\right)}^{2}}{V}}\;$$

where I_TEST_ is the test image, and I_LABEL_ is the corresponding label image. V denotes the number of voxels within the whole image. PSNR is mathematically expressed as follows:

(2) $$\mathrm{PSNR}\;=20\mathrm{lo}g_{10}\frac{I_{\max}}{\mathrm{RMSE}}$$

where I_max_ is the maximum intensity of the image. SSIM is mathematically expressed as follows:

(3) $$\mathrm{SSIM}\;=\frac{\left(2\mu_{x}\mu_{y}+C_{1}\right)\left(2\sigma_{\mathrm{xy}}+C_{2}\right)}{\left(\mu_{x}^{2}+\mu_{y}^{2}+C_{1}\right)\left(\sigma_{x}^{2}+\sigma_{y}^{2}+C_{2}\right)}\;$$

where μ_x_ and σ_x_ are the mean and standard deviation (SD) of I_TEST_, and μ_y_ and σ_y_ are the mean and SD of I_LABEL_. σ_xy_ denotes the covariance of I_TEST_ and I_LABEL_. C_1_ and C_2_ are small constants to stabilize the division with weak denominator.

**FIGURE 3:**
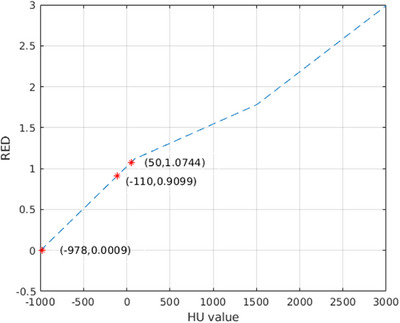
The calibration curve between hounsfield unit (HU) value and RED.

Because semantic segmentation methods can reduce the burden of manual contouring yet are often hindered by bias field artifacts, the second part of the evaluation compared the proposed workflow with the N4ITK algorithm to determine its impact on segmentation accuracy.^22^ In this study, the N4ITK implementation available in 3D Slicer was used.^23^ Image segmentation was carried out using the k‐means clustering algorithm with three clusters representing air, fat, and soft tissue.^24^ Segmentation accuracy was evaluated using the Dice similarity coefficient (DSC), which is mathematically expressed as follows:

(4) $$\text{DSC}=\frac{2\left|A\cap B\right|}{\left|A\right|\cap \left|B\right|}\times 100\%$$

where A is the binary mask of I_TEST_, and B is the binary mask of I_LABEL_. The three‐tissue segmentation masks from MR images were subsequently converted into relative electron density (RED) maps, with the air mask assigned an RED value of 0.0009, the fat mask assigned an RED value of 0.9099, and the water mask assigned an RED value of 1.0744. For comparison, RED maps were also generated from the corresponding CT images using the calibration curve presented in Figure 3. To evaluate the influence of bias field correction on electron density mapping, the percent difference in line integrated RED along a 180‐degree opposing beam was calculated between RED maps derived from MRI and those derived from CT. The mathematical expression of percent difference was shown as follows:

(5) $$\mathrm{percent}\;\mathrm{difference}\;=\frac{\left|\sum \mathrm{RE}D_{\mathrm{MRI}}-\sum \mathrm{RE}D_{\mathrm{CT}}\right|}{\sum \mathrm{RE}D_{\mathrm{CT}}}\times 100\%$$

where ΣRED_MRI_ and ΣRED_CT_ represent the line integrated RED along the ray path for RED maps converted from MRI and CT, respectively.

## 3 RESULTS

**FIGURE 4:**
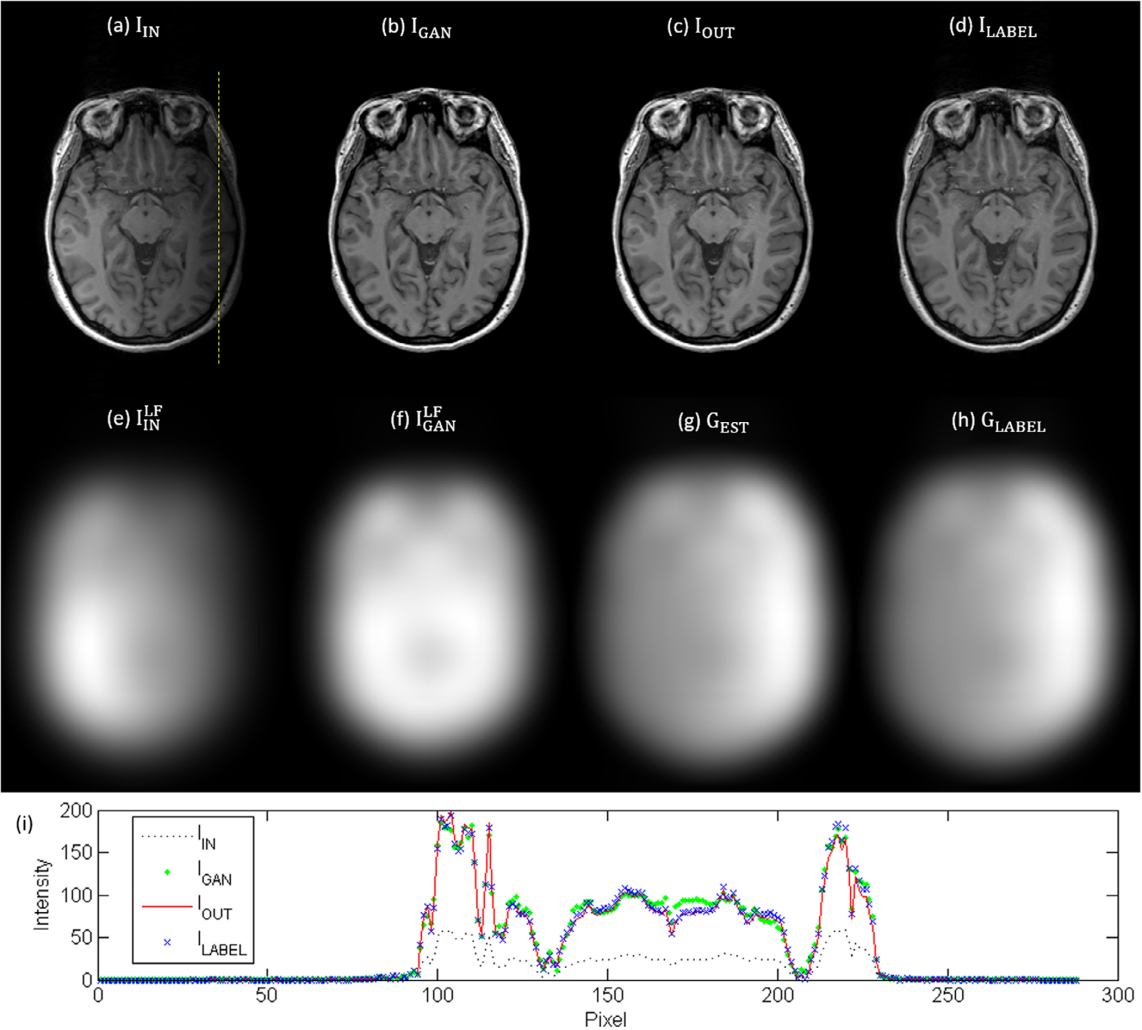
Example of (a) $I_{\mathrm{IN}}$, (b) $I_{\mathrm{GAN}}$, (c) $I_{\mathrm{OUT}}$, (d) $I_{\mathrm{LABEL}}$, (e) $I_{\mathrm{GAN}}^{\mathrm{LF}}$, (f) $I_{\mathrm{IN}}^{\mathrm{LF}}$, (g) $G_{\mathrm{EST}}$, (h) $G_{\mathrm{LABEL}}$ for T1‐weighted brain MR images and (i) intensity profiles along the yellow dashed line for $I_{\mathrm{IN}}$, $I_{\mathrm{GAN}}$, $I_{\mathrm{OUT}}$, $I_{\mathrm{LABEL}}$.

**FIGURE 5:**
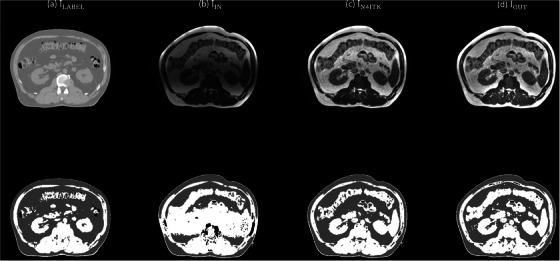
Axial images of (a) $I_{\mathrm{LABEL}}$, (b) $I_{\mathrm{IN}}$, (c) $I_{N4\mathrm{ITK}}$, (d) $I_{\mathrm{OUT}}$ (top row), and their 3‐tissue segmentation masks (bottom row) for patient with ID# 1ABB086.

**FIGURE 6:**
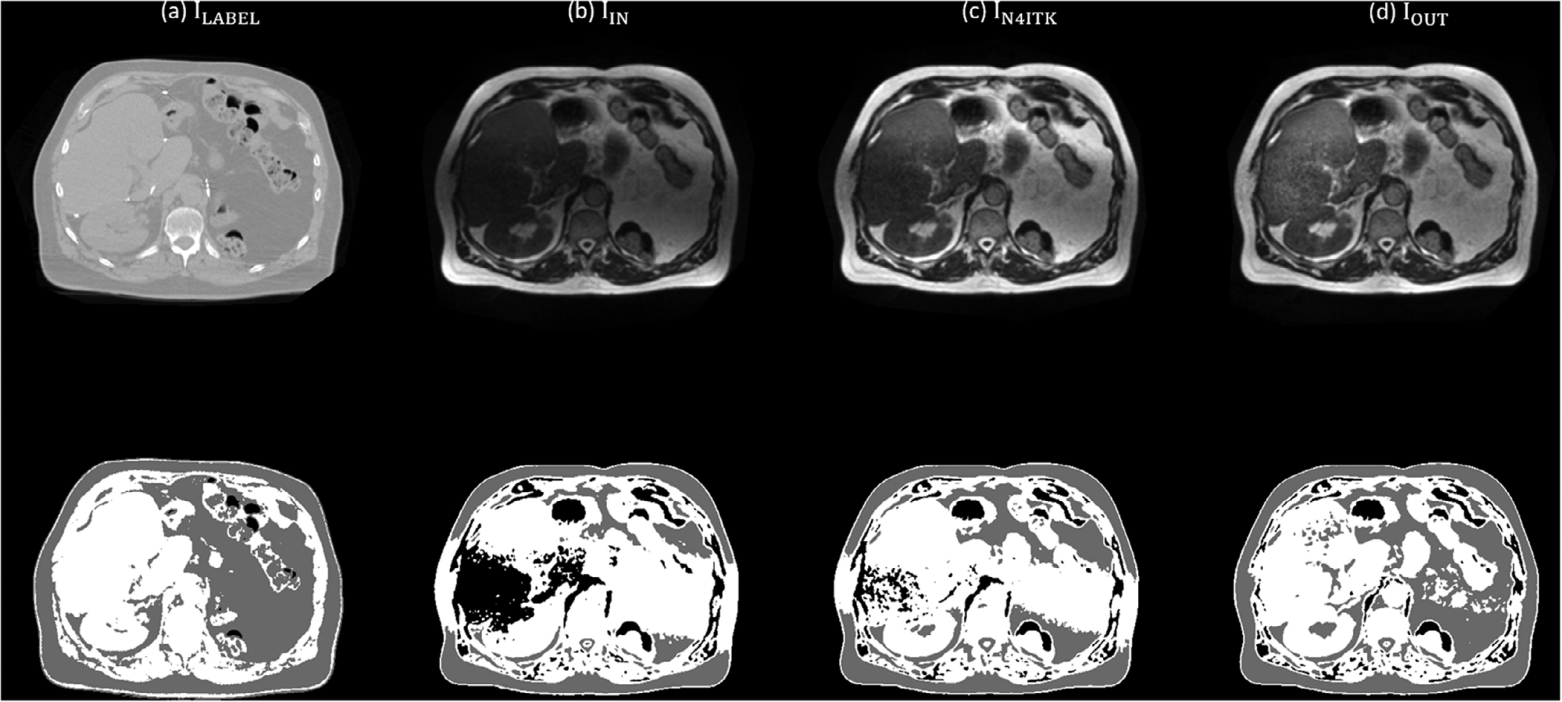
Axial images of (a) $I_{\mathrm{LABEL}}$, (b) $I_{\mathrm{IN}}$, (c) $I_{N4\mathrm{ITK}}$, (d) $I_{\mathrm{OUT}}\;$(top row), and their 3‐tissue segmentation masks (bottom row) for patient with ID# 1ABB098.

Figure 4 shows the T1‐weighted brain MR images corrected with N4ITK (I_LABEL_), I_LABEL_ corrupted with an inhomogeneity pattern not used during model training (I_IN_), the corrected images obtained using the GAN (I_GAN_) and the proposed workflow (I_OUT_), the low frequency components in I_IN_ ($I_{\mathrm{IN}}^{\mathrm{LF}}$) and I_GAN_ ($I_{\mathrm{GAN}}^{\mathrm{LF}}$), the estimated gradient map (G_EST_), the gradient map derived from I_LABEL_ (G_LABEL_), and intensity profiles along the yellow dashed line for I_IN_, I_GAN_, I_OUT_, I_LABEL_. Comparison of the intensity profiles shows that I_GAN_ exhibits a higher discrepancy than I_OUT_ when compared with I_LABEL_. The RMSE was 30.59, 12.06, 10.37 for I_IN_, I_GAN_ and I_OUT_, respectively. The PSNR was 42.34, 46.04, 47.04 dB for I_IN_, I_GAN_ and I_OUT_, respectively. The SSIM was 0.84, 0.96, 0.98 for I_IN_, I_GAN_ and I_OUT_, respectively. Table 2 presents a summary of the DSC results for abdominal scans acquired with 0.35T MR‐LINAC. The mean DSC of air segmentation masks was 0.95, 0.97 and 0.97 for I_IN_, I_N4ITK_ and I_OUT_, respectively. The mean DSC of fat segmentation masks was 0.61, 0.71 and 0.74 for I_IN_, I_N4ITK_ and I_OUT_, respectively. The mean DSC of soft tissue segmentation masks was 0.60, 0.68 and 0.69 for I_IN_, I_N4ITK_ and I_OUT_, respectively. The *P*‐values for air, fat and soft tissue segmentation masks were 0.0078, <0.001, <0.001, respectively, when comparing DSC values between I_IN_ and I_N4ITK_ using the Wilcoxon signed‐rank test at a 5% significance level. The corresponding *P*‐value were 0.0020, <0.001, <0.001 when comparing DSC values between I_IN_ and I_OUT_. Among the twelve patients included in this study, N4ITK outperformed the proposed workflow in four patients (ID# 1ABB066, 1ABB077, 1ABB084, 1ABB0086), whereas the proposed workflow achieved better results than N4ITK in the remaining eight patients. Figure 5 presents axial images for patient with ID# 1ABB086, including the CT image (I_LABEL_), the 0.35T MR image (I_IN_), the corrected images obtained using N4ITK (I_N4ITK_) and the proposed workflow (I_OUT_), along with their three‐tissue segmentation masks. Figure 6 shows the corresponding images and segmentation masks for patient with ID# 1ABB098. Table 3 summarizes the percent difference in line integrated RED along a 180‐degree opposing beam for RED maps converted from 0.35T MR images (I_IN_), the corrected images obtained using the GAN (I_GAN_) and the proposed workflow (I_OUT_). The mean values of percent difference were 9.20%, 3.84% and 2.30% for I_IN_, I_N4ITK_ and I_OUT_, respectively. Comparing the percent difference between I_IN_ and I_N4ITK_ using the Student's *t*‐test yielded a *P*‐value of 0.0069, whereas the comparison between I_IN_ and I_OUT_ resulted in a *P*‐value of 0.0010.

**TABLE 2:** The dice similarity coefficients for 0.35T MR images before and after bias field correction.

	I_IN_			I_N4ITK_			I_OUT_		
	Air	Fat	Soft tissue	Air	Fat	Soft tissue	Air	Fat	Soft tissue
1ABB002	0.90	0.67	0.63	0.94	0.73	0.66	0.93	0.73	0.68
1ABB024	0.93	0.59	0.65	0.93	0.66	0.66	0.94	0.72	0.69
1ABB036	0.99	0.52	0.83	0.99	0.58	0.83	0.99	0.59	0.84
1ABB066	0.93	0.64	0.59	0.97	0.78	0.71	0.96	0.76	0.70
1ABB073	0.97	0.58	0.57	0.97	0.60	0.58	0.98	0.68	0.62
1ABB077	0.95	0.71	0.52	0.98	0.82	0.70	0.98	0.81	0.70
1ABB084	0.93	0.63	0.43	0.96	0.80	0.61	0.97	0.80	0.60
1ABB086	0.98	0.58	0.59	0.99	0.73	0.65	0.99	0.71	0.64
1ABB098	0.93	0.47	0.55	0.95	0.56	0.65	0.96	0.69	0.71
1ABB117	0.93	0.68	0.62	0.97	0.75	0.72	0.97	0.75	0.73
1ABB150	0.99	0.64	0.69	0.99	0.71	0.71	0.99	0.78	0.72
1ABB151	0.93	0.64	0.48	0.97	0.82	0.69	0.97	0.85	0.71

**TABLE 3:** The percent difference (%) for 0.35T MR images before and after bias field correction.

	I_IN_	I_N4ITK_	I_OUT_
1ABB002	8.99	5.92	5.76
1ABB024	7.35	5.01	2.51
1ABB036	7.36	5.13	2.75
1ABB066	9.47	0.83	0.75
1ABB073	4.24	3.30	2.92
1ABB077	11.03	2.22	0.98
1ABB084	2.31	1.57	1.12
1ABB086	4.96	2.70	1.95
1ABB098	28.49	4.07	0.52
1ABB117	10.35	4.95	3.67
1ABB150	10.74	7.04	2.73
1ABB151	5.13	3.29	1.98

## 4 DISCUSSION

In recent years, several deep learning‐based approaches have been proposed for MRI bias field correction, each employing distinct network architectures and training strategies. Xu et al. introduced BiasNet, a framework for brain MR images built upon a deep separable convolutional neural network.^25^ Their method combined the original MR image with multiscale features extracted from bias field components, enabling the network to capture subtle intensity nonuniformities across multiple frequency bands. Simkó et al. proposed an alternative strategy using implicit training of convolutional neural networks (CNNs) to address intensity inhomogeneity, thereby eliminating the need for explicitly labeled training data and improving applicability to heterogeneous imaging scenarios.^26^ Chuang et al. advanced a GAN‐based framework capable not only of correcting coil inhomogeneity but also of automatically extracting the brain from both spin‐echo and gradient‐echo based echo‐planar imaging (EPI) scans, streamlining the preprocessing pipeline for functional neuroimaging.^27^ Sridhara et al. developed a convolutional autoencoder‐based model designed to directly predict the bias field in 3D MRI data, which improved intensity uniformity while preserving anatomical details, with performance surpassing N4ITK.^28^ While these methods have shown strong results, their generalizability has often been constrained by training on datasets acquired under controlled conditions, typically limited to specific scanners, field strengths, or pulse sequences.

To address this limitation, Kanakaraj et al. proposed DeepN4, a neural network approach based on a 3D U‐Net designed to replicate and accelerate N4ITK corrections for T1‐weighted brain MRI.^29^ Their training utilized eight independent cohorts spanning 72 scanners, covering diverse acquisition protocols and subject populations, thereby equipping the model to handle broad inter‐scanner and inter‐cohort variability. Experimental results demonstrated that DeepN4 closely matched N4ITK corrections, achieving high quantitative similarity, while enabling faster and more flexible deployment across imaging platforms. Compared with 1.5‐3T diagnostic MRI scanners, 0.35T MR‐LINAC systems are less common. Therefore, rather than relying on large training datasets, a bias field correction workflow was implemented to enhance the flexibility and generalizability of the GAN model. Bias field artifacts are generally less pronounced at lower magnetic fields due to reduced susceptibility effects.^30^, ^31^ Although bSSFP sequences are sensitive to field inhomogeneities, the 0.35T MR‐LINAC exhibits less off‐resonance banding than 3T systems.^32^, ^33^ The intensity variations in 0.35T abdominal MR images (see Figure 5(b) and 6(b)) are therefore more likely caused by surface coil sensitivity rather than field inhomogeneity, and the longer RF wavelength at 0.35T further reduces dielectric interference, contributing to spatial nonuniformities. There are now many automatic segmentation algorithms proposed for target delineation in radiotherapy.^34^, ^35^ Nonetheless, the generated contours must still be reviewed and refined by clinical staff, because image artifacts, anatomical variations, or algorithmic limitations may lead to misclassification or misinterpretation of structures. If such automated methods achieve sufficiently high accuracy, they have the potential to greatly reduce the workload on medical personnel while improving consistency and reproducibility of contours. Bias field correction using either N4ITK or the proposed workflow improved the DSC values for the segmentation masks between the test and label images, with statistically significant differences observed in all three tissue types at a significance threshold of *p* = 0.05. No statistically significant difference was observed when comparing the DSC values between I_N4ITK_ and I_OUT_ using the Wilcoxon signed‐rank test. Nevertheless, cases that exhibited poor correction performance with N4ITK (ID# 1ABB024, 1ABB073, 1ABB098, 1ABB150) showed noticeable improvement when processed using the proposed workflow. This enhancement also translated to more accurate percent differences in the line‐integrated RED along the ray path for RED maps derived from MRI compared with CT.

Since 2019, a 0.35T MR‐LINAC has been installed in our department. In non‐adaptive radiotherapy, the daily MRI is rigidly registered to the reference simulation image, and an isocenter shift is applied. Dose‐volume violations are only detectable if the dose is recalculated on the daily MRI, limiting this approach to cases with negligible anatomical changes. In online adaptive replanning, deformable registration is used to propagate reference contours of targets and OARs to the daily MRI. When significant anatomical changes are detected, a full replanning may be required to ensure accurate and safe dose delivery. By utilizing the daily image for optimization and dose calculation, this workflow accounts for anatomical changes, including variations in size, deformation, and the spatial relationships between targets and OARs. Adaptive radiotherapy is important for abdominal cancers, such as liver tumors, adrenal lesions and abdominal lymph node metastases, where inter‐fraction organ motion and variability can compromise tumor coverage and increase OAR exposure.^36^, ^37^ Accurate bias field correction of daily MRI enhances contour propagation and dose calculation by reducing intensity inhomogeneities that may otherwise degrade segmentation and registration. The proposed GAN‐based workflow enhances robustness and generalizability across different scanners and imaging conditions, facilitating safer and more precise adaptive radiotherapy for abdominal cancers using 0.35T MR‐LINAC.

## 5 CONCLUSION

In this study, a bias field correction workflow was proposed, which was designed to improve the flexibility and generalizability of the GAN model for abdominal cancer patients treated with a 0.35T MR‐LINAC system. Our results demonstrated that the workflow was effective for correcting not only brain MR images acquired on a 3T diagnostic scanner but also abdominal MR images obtained using a 0.35T MR‐LINAC system. By effectively mitigating bias field artifacts, the proposed workflow has the potential to strengthen the clinical utility of MRI‐guided adaptive radiotherapy for abdominal cancers, ensuring safer and more accurate radiation delivery.

## AUTHOR CONTRIBUTIONS

**Ching‐Ching Yang**: Conceptualization; methodology; software; validation; formal analysis; writing—review and editing; supervision. **Hung‐Te Yang**: Conceptualization; methodology; software; formal analysis; writing—review and editing.

## CONFLICT OF INTEREST STATEMENT

The authors have no competing interests to declare that are relevant to the content of this article.
